# Neutralizing monoclonal antibodies against SARS-CoV-2 for COVID-19 pneumonia in a rituximab treated patient with systemic sclerosis—A case report and literature review

**DOI:** 10.3389/fmed.2022.934169

**Published:** 2022-08-03

**Authors:** Melek Yalcin Mutlu, Jule Taubmann, Jochen Wacker, Koray Tascilar, Filippo Fagni, Maximilian Gerner, Daniel Klett, Georg Schett, Bernhard Manger, David Simon

**Affiliations:** ^1^Department of Internal Medicine 3, Friedrich-Alexander University (FAU) Erlangen-Nuremberg and Universitätsklinikum Erlangen, Erlangen, Germany; ^2^Deutsches Zentrum fuer Immuntherapie (DZI), FAU Erlangen-Nuremberg and Universitätsklinikum Erlangen, Erlangen, Germany; ^3^Department of Internal Medicine 1, FAU Erlangen-Nuremberg and Universitätsklinikum Erlangen, Erlangen, Germany

**Keywords:** neutralizing mAbs, systemic sclerosis, COVID-19, rituximab, casirivimab/imdevimab, immune mediated inflammatory disease

## Abstract

Patients with immune-mediated diseases (IMID) such as systemic sclerosis (SSc), who are treated with B cell depleting treatments, are at risk for developing severe COVID-19 due to inadequate humoral immune response. During B cell depletion, therapeutic substitution of neutralizing monoclonal antibodies against the SARS-CoV-2 spike protein (mAbs) might be helpful to prevent severe COVID-19. It has been shown, that in non-IMID patients mABs reduce SARS-CoV-2 viral load and lower the risk of COVID-19 associated hospitalization or death. However, there are limited data on the effect of mAbs in IMID patients after exposure, especially in patients treated with B cell depleting agents. Herein, we report a case of a rituximab treated SSc patient who developed COVID-19 and was successfully treated with a combination of mAbs (casirivimab/imdevimab). With this case we show that IMID patients may benefit from post-exposure administration of mAbs. In our case treatment with neutralizing autoantibodies was safe and a possible contributor in protecting the patient from mechanical ventilation and eventually death. We frame this case within the current evidence from the literature and provide a perspective on the future potential role of mAbs for treating IMID patients suffering from COVID-19.

## Introduction

B cell depleting therapies, such as rituximab (RTX), are widely used in patients with immune-mediated diseases (IMID), including Systemic Sclerosis (SSc). After RTX administration, circulating B cells rapidly decrease which leads to an impaired humoral immune response against infectious agents such as SARS-CoV-2. Therefore, RTX is associated with a higher risk of severe COVID-19 (1–3). During B cell depletion, therapeutic substitution of neutralizing monoclonal antibodies (mAbs) against the SARS-CoV-2 spike protein might be helpful to prevent severe COVID-19. Casirivimab/Imdevimab are two recombinant IgG1-mAbs that bind the receptor-binding domain of the SARS-CoV-2 spike protein and prevent virus binding to its receptor angiotensin-converting enzyme-2. Casirivimab/imdevimab reduce SARS-CoV-2 viral load and lower the risk of COVID-19 associated hospitalization or death (4). Here, we present a SSc-patient under treatment with RTX, who developed COVID-19 and was successfully treated with Casirivimab/Imdevimab. We also discuss this case along with similar cases reported so far with the aim to provide an overview and an outlook on the future potential role of mAbs for pre-exposure prophylaxis and post-exposure treatment of IMID patients.

## Case description

A 74-year-old woman was diagnosed with limited SSc in 2015. Concomitant diseases were arterial hypertension, hyperlipidemia and hypothyroidism. Her BMI was 32 kg/m^2^, she never smoked or consumed alcohol. She had no history of mental illness, lived alone and provided for herself. Initial disease manifestations were severe peripheral vasculopathy with Raynaud’s syndrome, recurrent digital ulcerations and inflammatory arthritis with positive RNA-polymerase-3 antibodies. In the past the patient didn’t suffer from dyspnea or cough. Lung and heart involvement were also ruled out by pulmonary function tests, high-resolution CT, electrocardiography, and cardiac MRI. She was initially treated with sildenafil, alprostadil, corticosteroids, and mycophenolate mofetil (MMF). Since 2017, MMF was discontinued and RTX was added due to persistent arthritis. For the last 2 years of treatment, during which CD19 + B cells were no longer detected, she was also free of digital ulcerations (Table 1).

**TABLE 1:** Demographic and clinical data of the patient.

Age, gender	74, female
Body mass index	32 kg/m^2^
Smoking habits/alcohol consumption	None
Comorbidities	Arterial hypertension Hyperlipidemia Hypothyroidism
Previous medications	Alprostadil (off-label use for severe vasculopathy) Corticosteroids Methotrexate Mycophenolate mofetil
Current medication	Rituximab 1,000 mg/every 6 months Sildenafil 20 mg/three times a day Valsartan + hydrochlorotiazide 160/12, 5 mg/per day Amlodipin 10 mg/per day L-thyroxin 100 μg/per day Colecalciferol 20,000 IU/twice a week
Allergies	Nitrendipine

The patient was vaccinated against SARS-CoV-2 6 months after the last RTX therapy. The first two mRNA vaccine BNT162b2 (Pfizer/Biontech) doses were administered in April and June 2021 with a 5-week interval. The patient didn’t develop any anti-SARS-CoV-2 antibody response. She was boostered early with the vector vaccine ChAdOx1 (AstraZeneca) in July 2021. Thereafter, she developed a moderate anti-SARS-CoV-2-IgG antibody response (OD450: 1.98; cut-off OD450: 0.8; Euroimmune ELISA) and a SARS-CoV-2 specific T-cell response in the IFN-γ-ELISpot assay.

In December 2021, 2 months after the last RTX administration, she presented to the emergency department with progressive dyspnea, fever and cough; her symptoms began 7 days earlier. There was no infected contact person to be traced. On physical examination she appeared ill and dyspneic with a body temperature of 38, 6°-Celsius and a respiratory rate of 21/min. Oxygen saturation was 97% in room air.

**FIGURE 1:**
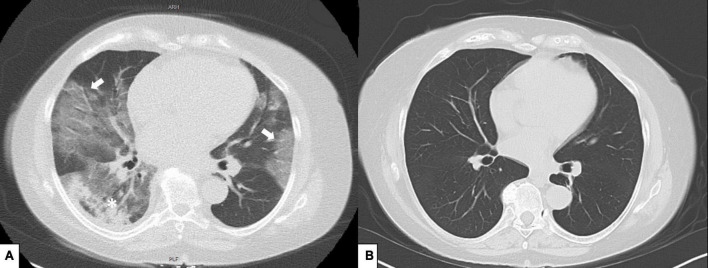
**(A)** Computed tomography scans with typical ground glass opacities (white arrows) and consolidation (asterix) predominantly on posterobasal regions, **(B)** control thorax tomography after 5 months.

Blood samples at the time of admission revealed lymphopenia (400 cells/μL), C-reactive protein was 143 mg/L (normal range < 5 mg/L), D-dimer 1,605 ng/mL (normal range < 500 ng/mL), ferritin 109 ng/mL (30–651 ng/mL), and IL-6 was 239.9 pg/mL (normal range < 7 pg/mL). Nasopharyngeal SARS-COV-2-PCR assay was positive and subsequent sequencing revealed infection with the Delta variant. At the time of admission, no SARS-COV-2 spike antibodies were detectable. CT-scans showed typical ground glass opacities consistent with COVID-19 pneumonia, the infiltration involved approximately 40% of the lung parenchyma (Figure 1A).

Blood and sputum cultures were obtained for the differential diagnosis of bacterial pneumonia, which was excluded based on the negative cultures and Gram staining. Pneumocystis carinii pneumonia was ruled out based on normal lactate dehydrogenase levels and imaging findings showing predominantly posterobasal segmental involvement.

The patient was hospitalized and inhaled symptomatic therapy (ipratropium bromide/salbutamol, oxygen) was initiated. Since she had several poor prognostic factors i.e., older age, concomitant hypertension, immunosuppression with a B cell depleting agent, high body mass index, low lymphocyte count, and lung involvement of 40%, she was considered at risk of mechanical ventilation and death. Therefore, treatment with mAbs was considered (1, 5) and Casirivimab/imdevimab (1,200 mg each) was administered. The administration was well tolerated and no immediate or late adverse event was observed during her admission and follow-up.

**FIGURE 2:**
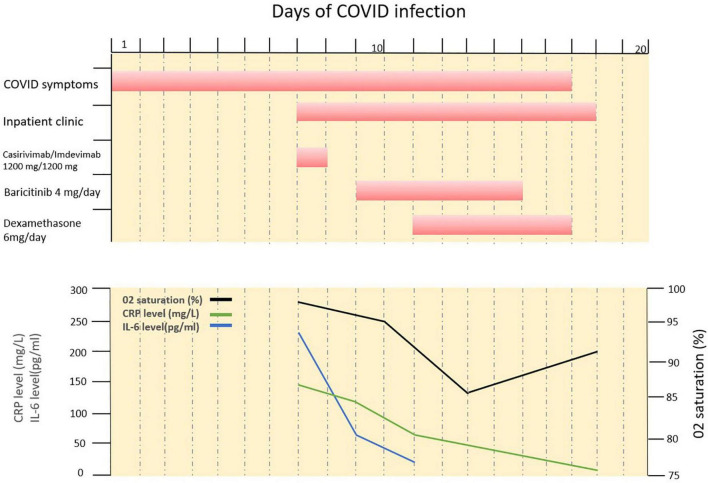
Time course of COVID symptoms and treatments, inflammation levels and peripheral oxygenation during COVID-19 infection. Day 0, COVID-19 symptoms started; Day 7, inpatient clinic + Casivirimab-imdevimab treatment; Day 9, transferred to COVID clinic-Baricitinib 4 mg added; Day 11, steroid treatment added; Day 18, discharge.

During the 3rd day of the hospital stay, oxygen demand increased and she was transferred to a specialized COVID ward for close monitoring. Patient’s saturation level dropped to a level of 91% (under 2 L/min oxygen). Baricitinib 4 mg/day was added and continued for 7 days. CRP level was 125 mg/L and IL-6 level 69 pg/mL. On the 5th day oxygen saturation further dropped to 85% (2 L/min oxygen). Dexamethasone 6 mg/day was initiated and continued for 7 days. After 7th day of hospitalization her clinical status started to improve, oxygen demand decreased and CRP level (10 mg/L) and IL-6 level (13 pg/mL) decreased (Figure 2). On the 11th day she was discharged from the hospital (Figure 2). After 2 months from her hospitalization, she fully recovered and was symptom-free. After 5 months her follow-up CT showed marked improvement (Figure 1B). Her SARS-CoV-2-spike IgG level was 10.3, reflecting the administered casirivimab/imdevimab.

## Discussion

We consider that the mAb therapy with casirivimab/imdevimab supported the recovery in this case by directly neutralizing the virus under a severely impaired humoral immune response against SARS-CoV-2. As COVID-19 pneumonia had already developed, the patient required additional inflammation control with glucocorticoids and baricitinib. Although it is not possible to dissect the exact contribution of casivibirimab/indevimab administration to the observed disease course in this very patient, our attempt at early viral load reduction with the use of monoclonal antibodies in a high-risk patient may have prevented a more severe course of hyperinflammation that would otherwise have been detrimental. We reason that monoclonal antibodies should work similar to the mechanism by which vaccination is better at preventing severe disease or death compared to preventing infection with SARS-CoV-2 (3, 4). Overall, administration of casirivimab/imdevimab was safe in our case.

Emerging variants such as Omicron have accumulated over 30 mutations in the spike protein. This poses a threat for the mAbs already licensed for clinical use as well as infection- and vaccine induced immunity. For this reason, detection of virus variants with subsequent sequencing plays a crucial role for treatment choice (6).

Anti-SARS-CoV-2 mAbs provide immune prophylaxis against COVID-19 and are the standard of care in patients who are at high risk for severe COVID-19 due to underlying disease or treatment. Currently, several mAbs such as bamlanivimab, bamlanivimab/etesevimab, casirivimab/imdevimab, cilgavimab/tixagevimab, and sotrovimab have been shown to be effective in reducing mortality and hospitalization rates in the general COVID patient population (7–11). While bamlanivimab, bamlanivimab/etesevimab, and sotrovimab can be used for post-exposure treatment, cilgavimab/tixagevimab can also be used for pre-exposure prophylaxis (12). On the other hand, studies showed that patients benefit from casirivimab/imdevimab both for prevention and treatment of COVID-19 infection (12, 13).

To date, there are only a modest number of case reports/series in the literature where mAbs for treatment of COVID have been used in IMID patients with or without B cell depleting agents (Table 2). Similar to our case, these cases also showed favorable outcomes with no safety concerns (13–17). Yetmar et al. (16) and McCreary et al. (17) have reported the largest case series of treatment with mAbs in immunocompromised patients. In the work of Yetmar et al., four different mAbs reduced hospitalization rates, mortality rates and persistent infections in 180 patients who received B cell depleting therapy (16). Of the 180 cases reported, majority were under treatment with rituximab for the treatment of a hematologic malignancy while a minority were IMID patients (*N* = 62). Unfortunately, no precise conclusion can be drawn from the data presented regarding the efficacy and safety of mAbs in the subgroup of IMID patients. McCreary and colleagues compared two routes of administration of casirivimab/imdevimab treatment, also including a subpopulation of 50 patients with rheumatoid arthritis. In this study, mAbs reduced the risk of hospitalization and death regardless of route of administration (17). Comparable results were observed in several other case reports (13–15) reporting 6 patients with various IMIDs (psoriasis, psoriatic arthritis, Sjögren’s syndrome/Raynaud disease, and systemic lupus erythematosus). One study assessed breakthrough infection and the effect of post-exposure therapy in IMID patients treated with B-cell-depleting drugs. Of the 1,696 IMID patients included, 74 developed COVID-19 breakthrough infections, of whom 21 were subsequently treated with mAbs (casirivimab/imdevimab). Treatment with mAbs was associated with favorable clinical outcomes, with only one patient requiring hospitalization and no adverse outcomes (mechanical ventilation/death) observed (18).

**TABLE 2:** Overview of current evidence for pre-exposure prophylaxis or post-exposure treatment of mABs in IMID patients.

IMID	N cases	Pre-exposure prophylaxis or post-exposure treatment	Immunomodulatory background treatment	mAbs against SARS-CoV-2	References
Sjögren’s syndrome and Raynaud disease	1	Post-exposure treatment	Conservative management	Casirivimab + imdevimab	(13)
Psoriatic Arthritis	1	Post-exposure treatment	Ixekizumab	Casirivimab + imdevimab	
Psoriasis	1	Post-exposure treatment	Topical treatments	Bamlanivimab	
Systemic Lupus Erythematosus	1	Post-exposure treatment	Mycophenolate mofetil	Bamlanivimab	
Multiple sclerosis	1	Post-exposure treatment	Ocrelizumab	Casirivimab + imdevimab	(14)
Multiple sclerosis	1	Post-exposure treatment	Ocrelizumab	Casirivimab + imdevimab	(15)
Rheumatologic disorder*	62	Post-exposure treatment	Rituximab	Bamlanivimab Bamlanivimab + etesevimab Casirivimab + imdevimab Sotrovimab	(16)
Rheumatoid arthritis	50	Post-exposure treatment		Casirivimab + imdevimab	(17)
Various IMID*	21	Post-exposure treatment	Prednisone Methotrexate Azathioprine Mycophenolate mofetil	Casirivimab + imdevimab	(18)
2 Systemic Lupus Erythematosus, 1 Immune cytopenia, 1 Systemic sclerosis, 2 Immune encephalitis, 1 Myositis, 3 Systemic vasculitis	10	Pre-exposure prophylaxis	B-cell depleting therapy Azathioprine Cyclophosphamide Mycophenolate mofetil Bruton’s tyrosine kinase (BTK) inhibitor	Tixagevimab + cilgavimab	(19)
8 Granulomatosis Polyangiitis, 6 Rheumatoid Arthritis, 2 Microscopic Polyangiitis, 1 Systemic Sclerosis, 1 Systemic Lupus Erythematosus, 1 Multiple sclerosis, 1 CVID** 1 uveitis, 1 IgG4-related disease	22	Pre-exposure prophylaxis	Rituximab	Casirivimab + imdevimab	(20)

mAbs, monoclonal antibodies; SARS-CoV2, severe acute respiratory syndrome coronavirus-2; IMID, immune mediated inflammatory disease; CVID, common variable immune deficiency.

*Not clearly specified in publication.

**Common variable immune deficiency patient with granulomatous and lymphocytic interstitial lung disease.

Regarding pre-exposure prophylaxis in IMID, the current evidence is even more limited. To date, there has been only two studies examining the approach of using mAbs to protect patients from developing COVID-19. One of the studies examined whether IMID patients with B-cell depleting therapy who were fully vaccinated but did not show an adequate humoral response benefit from pre-exposure prophylaxis (19). Ten IMID patients with complete SARS-CoV-2 vaccination and inadequate humoral response were administered mAbs (tixagevimab/cilgavimab). The patients receiving a pre-exposure administration of mAbs had a lower risk of COVID-19. In another study we showed that casirivimab/imdevimab prophylaxis rapidly provides anti-SARS-CoV-2 humoral immunity in B cell depleted IMID patients who failed previous COVID-19 vaccination (20). These results indicate that IMID patients with B-cell depletion could benefit from prophylactic administration of mAbs after assessment of humoral response to SARS-CoV-2 vaccination (21, 22) and adapted vaccination regimens as necessary (22), in order to prevent the development of COVID-19 (19).

In summary, the case we have presented, as well as current evidence from the literature, demonstrates that anti-SARS-CoV-2 mAbs are a useful therapeutic option in IMID patients with or without B-cell-depleting agents who develop COVID-19. Further data on the efficacy of using mAbs for pre-exposure prophylaxis are urgently awaited, and a continuous reassessment of the benefits is needed over time due to changing dominant variants of the virus.

## Data availability statement

All data are presented in the case description.

## Ethics statement

Written informed consent was obtained from the individual(s) for the publication of any potentially identifiable images or data included in this article.

## Author contributions

MYM, KT, FF, BM, and DS: conceptualization, methodology, and writing—original draft preparation. MYM, JT, JW, KT, FF, MG, DK, GS, BM, and DS: writing—review and editing. All authors have read and agreed to the submitted version of the manuscript.
